# The Ras GTPase-Activating Protein Rasal3 Supports Survival of Naive T Cells

**DOI:** 10.1371/journal.pone.0119898

**Published:** 2015-03-20

**Authors:** Ryunosuke Muro, Takeshi Nitta, Toshiyuki Okada, Hitoshi Ideta, Takeshi Tsubata, Harumi Suzuki

**Affiliations:** 1 Department of Immunology and Pathology, Research Institute, National Center for Global Health and Medicine, Ichikawa-shi, Chiba, Japan; 2 Department of Immunology, Medical Research Institute, Tokyo Medical and Dental University, Bunkyo-ku, Tokyo, Japan; University of Tokyo, JAPAN

## Abstract

The Ras-mitogen-activated protein kinase (MAPK) pathway is crucial for T cell receptor (TCR) signaling in the development and function of T cells. The significance of various modulators of the Ras-MAPK pathway in T cells, however, remains to be fully understood. Ras-activating protein-like 3 (Rasal3) is an uncharacterized member of the SynGAP family that contains a conserved Ras GTPase-activating protein (GAP) domain, and is predominantly expressed in the T cell lineage. In the current study, we investigated the function and physiological roles of Rasal3. Our results showed that Rasal3 possesses RasGAP activity, but not Rap1GAP activity, and represses TCR-stimulated ERK phosphorylation in a T cell line. In systemic Rasal3-deficient mice, T cell development in the thymus including positive selection, negative selection, and β-selection was unaffected. However, the number of naive, but not effector memory CD4 and CD8 T cell in the periphery was significantly reduced in Rasal3-deficient mice, and associated with a marked increase in apoptosis of these cells. Indeed, survival of Rasal3 deficient naive CD4 T cells *in vivo* by adoptive transfer was significantly impaired, whereas IL-7-dependent survival of naive CD4 T cells *in vitro* was unaltered. Collectively, Rasal3 is required for *in vivo* survival of peripheral naive T cells, contributing to the maintenance of optimal T cell numbers.

## Introduction

T cells develop from their most immature CD4^-^ CD8^-^ double negative (DN) into CD4^+^ CD8^+^ double positive (DP) cells through β-selection in the thymus. Each DP cell expresses a T cell receptor (TCR) of different antigen specificity that is positively or negatively selected by interaction with major histocompatibility complex (MHC) / self-peptide complexes expressed by thymic epithelial cells. DP cells are selected for survival through relatively weak TCR stimulation (positive selection) and develop into class II MHC-restricted CD4 single positive (CD-4SP) cells or class I MHC-restricted CD8 single positive (CD8-SP) cells. In contrast, DP cells expressing self-reactive TCRs undergo apoptosis induced by strong TCR stimulation (negative selection) [1]. Because selection is mediated by TCR/peptide-MHC ligation, TCR-dependent signal transduction is critical for these selection events. Indeed, many of the signaling components in this pathway have been shown to be mandatory for selection.

TCR-signaling is also important for survival of mature naive T cells in the periphery [2]. It is known that the survival of CD44^lo^ CD62L^hi^ naive T cells requires self-peptide-MHC-induced weak continuous TCR signaling, accompanied by cytokine signaling such as IL-7 or IL-15 [3]. This weak, so-called tonic, TCR signaling is presumed to be below the threshold required to activate naive T cells [3]. Various studies have shown that interaction of TCR with self-peptide class I MHC is indispensable for cell survival of naive CD8 T cells [4–5]. In the case of CD4 T cells, long-term survival of naive CD4 T cells in the periphery similarly requires self-peptide class II MHC interactions [6–7], although some results have argued against this [8–9]. Besides TCR-induced signaling, it is well known that IL-7 and IL-15 are important for cell survival in the periphery by inducing anti-apoptotic genes such as Bcl2, in addition to down-regulating genes related to apoptosis [10–11].

The small G-protein Ras is a critical regulator of the mitogen-activated protein kinase (MAPK) pathway, which is an important component in TCR-mediated signal transduction [12]. The Ras-MAPK pathway is required for β-selection [13] and positive selection [14] in the thymus, as well as for proliferation, cytokine production and effector differentiation of peripheral mature T cells [12]. Ras activity is regulated positively and negatively by guanine nucleotide exchange factors (GEF) and GTPase-activating proteins (GAP), respectively. Therefore, these modulators of Ras activity are important in TCR-mediated signal transduction. RasGRP1, a RasGEF expressed in thymocytes, is essential for positive selection [12], whereas SOS1/2, another well-studied GEF, seems dispensable for T cell development [15]. Less well established is the significance of RasGAPs in T cell signaling. More than 10 different RasGAPs have been identified in mammals, and their biological significance was investigated by using their gene knockout mice [16]. Regarding their roles in T cells, only two of them have been reported. Rasa1 regulates positive selection negatively [17], whereas Neurofibromin 1 (NF1) regulates positive selection positively [18]; results that are in opposition. Therefore, the function of RasGAPs in T cell signaling remains unresolved.

We have previously identified functionally uncharacterized genes predominantly expressed in the thymus by in silico cloning, and have promoted study about two of these gene, Themis [19] and RhoH [20]. Ras-activating protein-like 3 (Rasal3) was another member of these genes, and it belongs to the SynGAP family [16], containing a Pleckstrin-homology (PH) domain, C2 domain and RasGAP domain. In the current study, we demonstrated that Rasal3 possessed RasGAP activity. Therefore, Rasal3 is another novel RasGAP expressed in T lineage cells. Our results with Rasal3-deficient mice revealed that Rasal3 is dispensable for T cell development in the thymus but is required for survival of naive T cells in the periphery, suggesting possible involvement of Rasal3 in tonic TCR signaling in the periphery.

## Materials and Methods

Animal experiments were approved by the Animal Care and Use Committee of the National Center for Global Health Medicine (NCGM) Research Institute and conducted in accordance with institutional procedures.

### Mice

C57BL/6 mice were purchased from SLC Japan (Shizuoka, Japan). OT-I-TCR-transgenic mice, OT-II-TCR-transgenic mice, HY-TCR transgenic mice, Rag2^-/-^ mice and CD45.1 mice were previously reported [21–22]. To generate Rasal3-deficient mice, a targeting vector with exons 6–9 of Rasal3 flanked by loxP sequences was constructed and electroporated into C57BL/6-derived embryonic stem (ES) cells. Recombinant ES cells were used to generate chimeric mice that were then mated with C57BL/6 mice to confirm germline transmission. The Rasal3-floxed mice were crossed with CAG-Cre mice, resulting in the deletion of exons 6–9 of Rasal3, which causes a frameshift at amino acid (aa) 292 with premature termination of translation at aa 306. PCR analysis demonstrated the successful deletion of the genomic region containing the exons 6–9, and Western blot analysis revealed the loss of Rasal3 protein expression in thymocyte lysate. All mice were bred and maintained under specific pathogen-free conditions in our animal facility. Mice were sacrificed by cervical dislocation to dissect organs out. All animal experiments and procedures were approved by the Animal Care and Use Committee of the National Center for Global Health Medicine Research Institute and conducted in accordance with institutional procedures.

### Quantitative RT-PCR

Total RNA was isolated from tissues or sorted cells by using the RNeasy kit (Qiagen) and reverse-transcribed with SuperScript III (Invitrogen). Real-time PCR was performed with Platinum SYBR Green qPCR-UDG Supermix with ROX (Invitrogen). Results were normalized to β-actin expression levels.

### Cell culture and retrovirus infection

Primary T cells and cell line DPK [19] were cultured in RPMI-1640 complete medium [23] containing 10% fetal calf serum at 37°C. A cDNA fragment encoding Rasal3 was inserted into the retrovirus vector pMRX-IRES-EGFP [24]. For retrovirus infection of DPK cells, Plat-E packaging cells were co-transfected with the retrovirus plasmid and VSV-G expression plasmid pMD.G as described previously [23]. Culture supernatants containing VSV-G-enveloped retrovirus were used for infection of DPK cells in the presence of 10 μg/ml polybrene.

### TCR stimulation

For stimulation of cell line DPK, the cells were incubated at 37°C for 5 min. The incubated cells were then mixed with equal volume of RPMI-1640 complete medium containing biotin-conjugated anti-CD3ε (145–2C11, Biolegend 50μmg/ml), anti-CD4 (GK1.5, Biolegend 50 μg/ml) and streptavidin (BECKMAN COULTER, 10 μg/ml) at 37°C for 1 min. Ice-cold PBS was added to stop stimulation.

### Western blot analysis

Cells were lysed with lysis buffer (50 mM Tris-HCl [pH7.5], 150 mM NaCl, 10 mM MgCl_2_, 0.5% Nonidet P-40) containing protease and phosphatase inhibitor cocktail (Thermo Scientific). Antibodies used for Western blotting are as follows: anti-phospho-ERK (197G2, Cell Signaling) anti-Ras (Ras-10, Millipore) anti-Rasal3 (homemade poly-clonal Rabbit IgG). Horseradish peroxidase-conjugated anti-IgG secondary antibodies against rabbit or mouse IgG (GE) were used with Lumiglo substrate (Cell signaling).

### RasGAP and RapGAP assay

Cells were lysed with lysis buffer containing 10% (w/v) glycerol. Ras Assay Reagent (Raf-1 RBD, agarose, Millipore) or Rap1 Assay Reagent (Ral GDS-RBD, agarose, Millipore) was added to the cell lysate and incubated at 4°C for 30 min. The precipitates were washed with the same lysis buffer for 3 times and subjected to Western blot analysis.

### Flow cytometry

For cell-surface staining, the following Antibodies were used: anti-CD4 (RM4–5 and GK1.5), anti-CD8α 5H10–1 and 53–6.7), anti-TCRβ (H57–597), anti-CD44 (IM7), anti-CD62L (MEL-14), anti-CD2 (RM-25), anti-CD5 (53–7.3), anti-CD69 (H1.2F3), anti-CD25 (PC61), anti-CD45.2 (104). For detection of apoptotic cells, cells were stained with FITC-conjugated Annexin V and 7-aminoactinomycin D (7AAD).

### Subcellular Protein Fractionation

Nuclear and cytoplasmic fractions were isolated from total thymocytes using the Subcellular Protein Fractionation Kit (Pierce) following the manufacturer’s protocol.

### IL-2 measurement

Sorted naive CD4 T cells were cultured in RPMI-1640 complete medium with immobilized anti-CD3ε The supernatant was used for Mouse IL-2 ELISA Ready-SET-Go! (eBioscience) according to the manufacturer’s protocol.

### Calcium flux measurement

Ca^2+^ flux was measured by flow cytometry. Thymocytes or splenocytes were rested for 30 min at 37°C. Cells from Rasal3-deficient mice were labeled with 5nM of carboxyfluorescein diacetate, succinimidyl ester (CFSE). Labeled and unlabeled cells were mixed at a ratio of 1:1 and were treated with Indo-1, AM (Life Technologies) for 30 min at 37°C. Then the cells were washed and stained with CD4 and CD8. The cells were pre-warmed for 5min at 37°C before assay. Anti-CD3ε (5 μg/ml) plus anti-CD28 (5 μg/ml) and ionomycin (1 μg/ml) were added into the cell suspension.

### Statistical Analysis

All data are represented as means ± SEM. Flow cytometric data were analyzed using the Unpaired T test (GraphPad Prism version 5.0). Asterisks in all figures are as follows: *P<0.05; **P<0.01. n.s.; not significant.

## Results

### Rasal3 was expressed in T cells and functioned as a RasGAP

**Fig 1:**
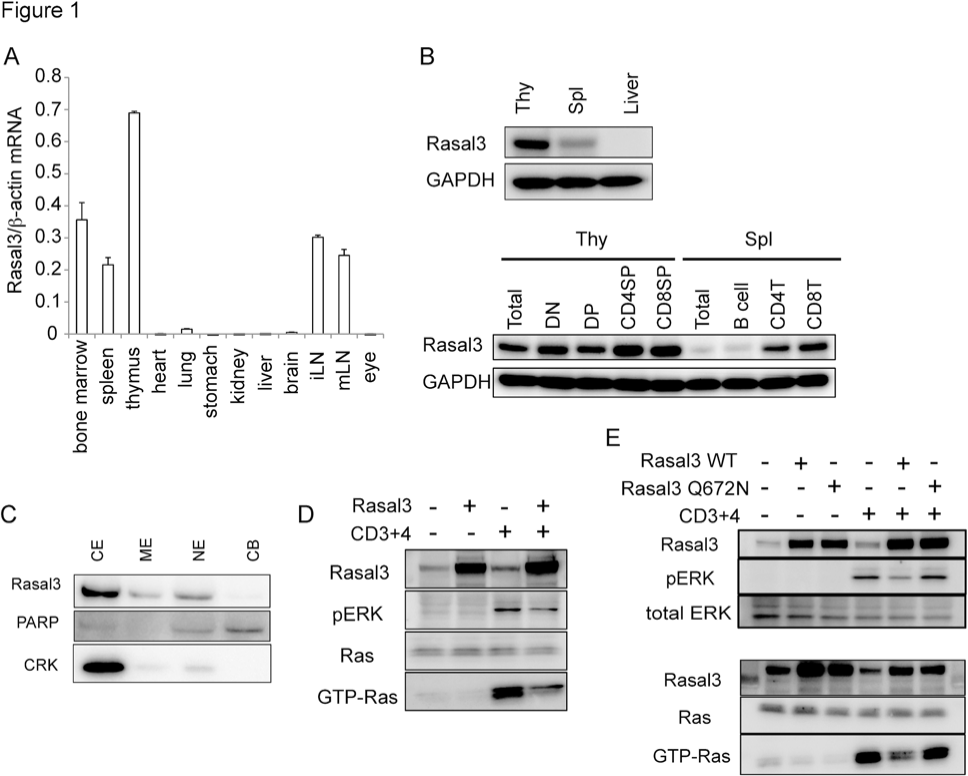
Expression, localization and function of Rasal3. (A) Expression of Rasal3 mRNA in various organs from C57L/6 mice. Rasal3 mRNA expression was determined by quantitative RT-PCR and normalized relative to β-actin mRNA. (B) Expression of Rasal3 protein in the thymus, spleen, and liver (upper) and sorted thymic or splenic T cell or B cell subpopulations (lower). (C) Subcellular localization of Rasal3 protein in thymocytes. CE, ME, NE, and CB indicate cytoplasmic extract, membrane extract, nuclear extract, and chromatin bound, respectively. (D) GAP activity of Rasal3. DPK cells overexpressing Rasal3 were subjected to a Ras-GTP pull-down assay followed by Western blot analysis. (E) The Q672N mutant Rasal3 could not suppress the increase in GTP-Ras and ERK activation. All data are representative of more than three independent experiments.

To identify novel genes specifically expressed in the thymus, we used our own algorithm to *in silico* clone several genes from the NCBI Unigene database of expressed sequence tags (ESTs) and *Rasal3* was one of the genes. As predicted, expression of Rasal3 mRNA was restricted to lymphoid tissues, and was highest in the thymus (Fig. 1A). Rasal3 protein expression in the thymus was confirmed by Western blot (Fig. 1B). Along thymocyte development, its expression was decreased in CD4 CD8 double positive cells (DP) then peaked in CD4-single positive (SP) and CD8-SP. In the periphery, expression was decreased again in mature T cells. Expression was not detected in B cells. By subcellular fractionation, Rasal3 protein was concentrated in the cytoplasmic fraction, indicating that it is cytoplasmic protein (Fig. 1C). Since Rasal3 contains a RasGAP domain, we next investigated its RasGAP activity. The DP thymoma line DPK was transduced with Rasal3 cDNA, which resulted in 10-fold greater Rasal3 protein expression. Overexpressing cells were then measured for TCR-dependent Ras-activation. The overexpression of Rasal3 strongly diminished TCR-induced activation of Ras, indicating that Rasal3 indeed possesses RasGAP activity (Fig. 1D). Consistent with this, TCR-dependent activation of ERK, which is a downstream event of Ras-activation, was also impaired in Rasal3-transduced DPK. Moreover, introduction of the Q672N mutation in RasGAP domain canceled the suppressive effect of Rasal3 in TCR-induced Ras activation and its downstream ERK activation (Fig. 1E), confirming that Rasal3 possesses RasGAP activity. It is known that some RasGAPs such as Rasa3 have not only RasGAP activity but also Rap1GAP activity [25], therefore we analyzed the Rap1GAP activity of Rasal3. However, in Rasal3-transduced DPK the amount of stimulation-dependent GTP-bound Rap1 was not affected (S1B Fig.), indicating that Rasal3 has no Rap1GAP activity.

### T cell development was not affected in the absence of Rasal3

**Fig 2:**
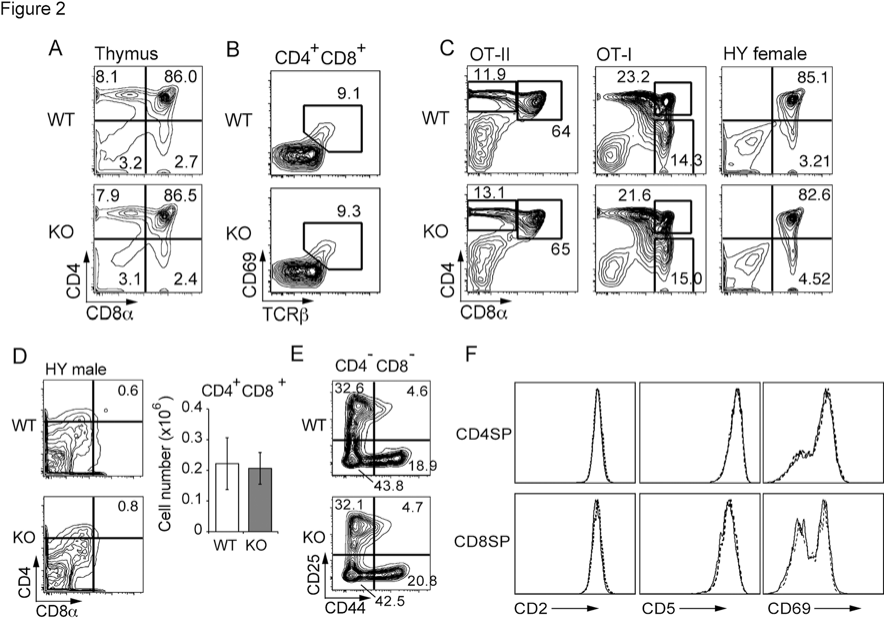
T cell development in Rasal3-deficient mice. (A) Flow cytometry profiles for CD4 and CD8 of thymocytes from WT and Rasal3-KO mice. (B) Proportions of TCRβ^+^ CD69^+^ post-selected cells in DP thymocytes. (C) Flow cytometry profiles for CD4 and CD8 of thymocytes from OT-II, OT-I, and female HY TCR-Tg RAG2^-/-^ mice. (D) Flow cytometry profiles for CD4 and CD8 of male HY Tg RAG2^-/-^ thymocytes (left). The bar graph shows the number of DP thymocytes (n = 3–4, mean ± s.e.) (right). (E) Analysis of developmental stage of DN thymocytes. Representative cytometry profiles for CD25 and CD44 expression in DN thymocytes are shown. (F) Expression of differentiation makers (CD2, CD5 and CD69) on CD4-SP or CD8-SP thymocyte. Solid and dashed lines indicate WT and Rasal3-KO, respectively.

To clarify the physiological role of Rasal3, we generated Rasal3-floxed mice to excise exon 6 to exon 9 of the gene. Breeding these floxed mice with CAG-Cre mice followed by removing the Cre-trangene resulted in systemic Rasal3 deficient mice. As shown in S2C Fig., Rasal3 protein in the T cells from Rasal3 deficient mice was barely detected by the Western blotting. In thymi of Rasal3 deficient mice, total cell number and frequency of CD4 CD8 double negative (DN), DP, CD4-SP and CD8-SP were not altered (Fig. 2A, S3A Fig.). Consistently, the proportion of immediate post-selected CD69^+^ DP cells was not changed in these mice (Fig. 2B). Rasal3 deficient mice were then further bred with OT-II, OT-I and HY TCR-transgenic (tg) mice with Rag2 deficiency to analyze the precise effects of Rasal3 on positive selection. The results demonstrated that positive selection of these three different monoclonal TCRs was not affected (Fig. 2C, S3B–D Fig.). The effect of Rasal3 on negative selection was negligible, as evaluated by the male HY TCR Tg system [26] (Fig. 2D). In addition, β-selection, evaluated by the ratio of DN3 (CD44^lo^ CD25^hi^) to DN4 (CD44^lo^ CD25^lo^) (Fig. 2E), and maturation of single positive thymocytes, evaluated by expression level of differentiation markers such as CD2, CD5, CD69 (Fig. 2F) were not changed in the deficient mice. Collectively, we concluded that development of conventional TCRαβ T cells in the thymus was independent of Rasal3. Additionally, development of unconventional T cells such as TCRγδ T cells and Tregs was normal as well (data not shown).

### Anti-TCR antibody-induced signal transduction was not enhanced in the absence of Rasal3

**Fig 3:**
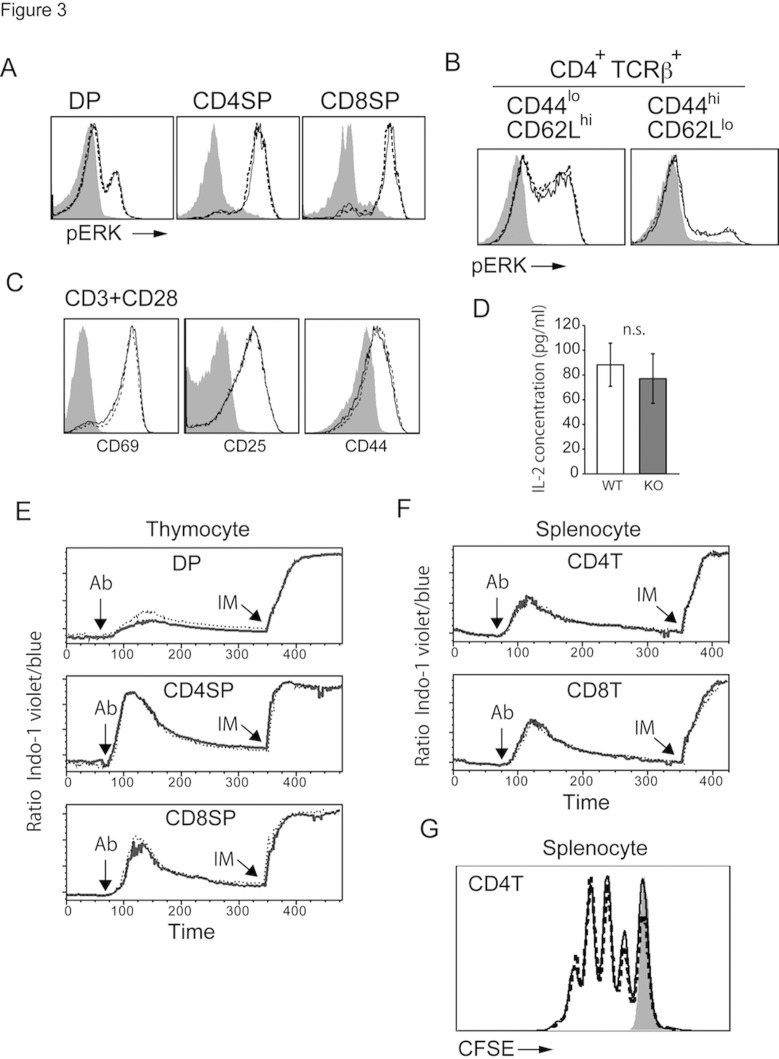
Rasal3 deficient T cells were activated normally upon agonistic anti-TCR antibody stimulation. TCR-induced phosphorylation of ERK in thymocytes (A) and splenocytes (B). (A) Total thymocytes were stimulated with anti-CD3 Ab (5 μg/ml) for 1 min. (B) Total splenocytes were stimulated with anti-CD3 (30 μg/ml) and CD28 (12 μg/ml) Abs for 1min. (C) Upregulation of CD69, 25, and 44 upon stimulation. Sorted naive CD4 T cells (CD4^+^ CD44^lo^ CD62L^hi^ CD25^-^) were activated by immobilized anti-CD3 Ab (5 μg/ml) and soluble anti-CD28 Ab (1 μg/ml) for 24 hours. Expression of CD69, CD25 and CD44 were analyzed by FACS. (D) IL-2 production from naive CD4 T cells. The cells were activated by immobilized CD3 (5 μg/ml) for 24 hours and IL-2 production was measured by ELISA. (E) and (F) Calcium influx triggered by TCR stimulation. Anti-CD3 plus anti-CD28 (Ab) and ionomycin (IM) were added at the points indicated by arrows. (G) CFSE-labeled splenic CD4 T cells were activated by immobilized anti-CD3 Ab (5 μg/ml) and soluble anti-CD28 Ab (1 μg/ml) for 72 hours. CFSE dilution by cell division was measured by flow cytometry. These data are representative of more than three experiments. Solid and dashed lines indicate WT and Rasal3-KO, respectively. The shadow indicates non-stimulated cells as a control.

Because RasGAPs negatively regulate Ras activation, loss of a RasGAP would promote augmentation of TCR-dependent signal transduction. Therefore, we examined the effects of Rasal3 deficiency on T cell signaling. The ligation of TCR by anti-TCR antibody induced Ras-dependent activation of ERK in thymocytes, splenic naive and memory CD4 T cells. As shown in Fig. 3A and B, antibody-stimulated activation of ERK was unchanged in Rasal3 deficient T cells. Anti-TCR antibody- induced upregulation of CD69, CD25, and CD44 was normal in the absence of Rasal3 (Fig. 3C). Furthermore, TCR-dependent proliferation, IL-2 production, and Ca^2+^ influx were all normal in Rasal3 deficient T cells (Fig. 3D–G). Collectively, the lack of Rasal3 did not enhance TCR-mediated signal transduction upon stimulation with anti-TCR antibody, possibly because of compensatory effects of other RasGAPs in T cells.

### Rasal3-deficient peripheral naive T cells were decreased with increased apoptosis

**Fig 4:**
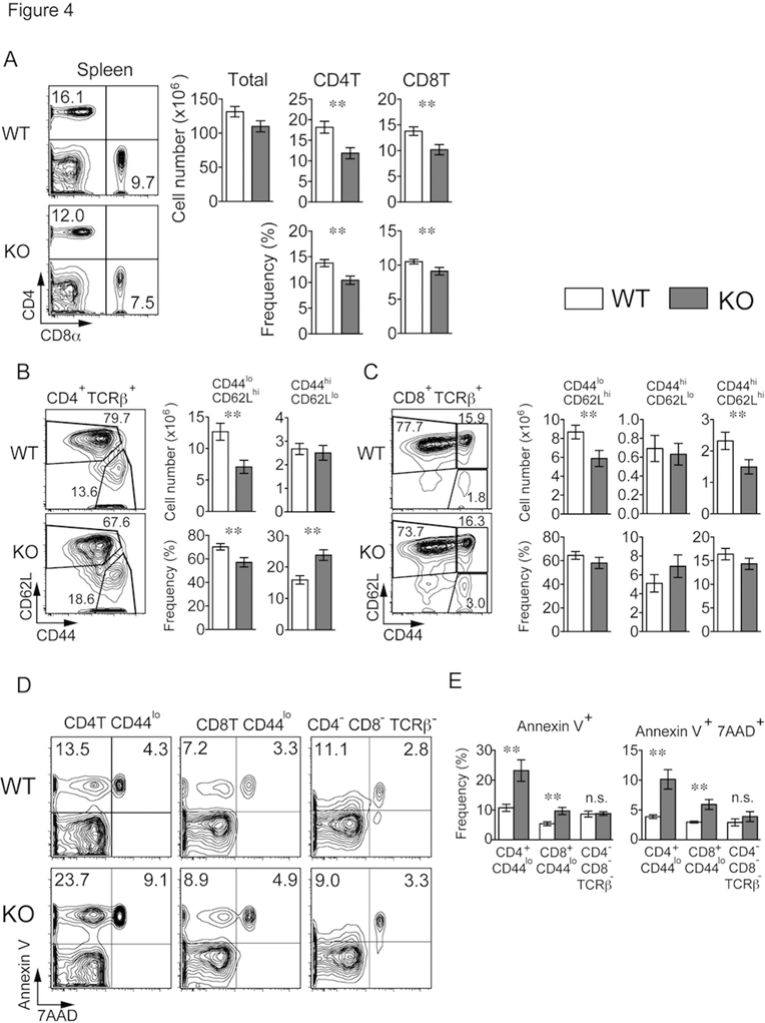
Decreased naive CD4 and CD8 T cells with increased apoptosis in Rasal3-KO mice. (A) Flow cytometry profiles for CD4 and CD8 of splenocytes from WT and Rasal3-KO mice (left). The bar graphs show the number of total splenocytes and number and frequency of splenic CD4^+^ TCRβ^+^ and CD8^+^ TCRβ^+^ cells (n = 19 mean ± s.e.) (right). (B and C) Profiles of CD44 and CD62L in splenic CD4^+^ TCRβ^+^ (B) or CD8^+^ TCRβ^+^ (C) cells (left). The bar graphs show the number and frequency of naive (CD44^lo^ CD62L^hi^) cells, effector memory (CD44^hi^ CD62L^lo^) cells and central memory (CD44^hi^ CD62L^hi^) cells (n = 17, mean ± s.e.) (right). (D) Annexin V and 7AAD staining of CD4^+^ TCRβ^+^ CD44^lo^, CD8^+^ TCRβ^+^ CD44^lo^ and CD4^-^ CD8^-^ TCRβ^-^ spleen cells freshly isolated from WT or Rasal3-KO mice. (E) The bar graphs show the frequency of Annexin V^+^ 7AAD^-^ and Annexin V^+^ 7AAD^+^ cells depicted in panel D (n = 5, mean ± s.e.).

Although T cell development in Rasal3-deficient thymus was completely normal, we found that the absolute number and frequency of mature CD4 and CD8 T cells in the spleen as well as in the inguinal lymph node (iLN) and peripheral blood were significantly reduced (Fig. 4A, S4A–B Fig.). The numbers of naive (CD44^lo^ and CD62L^hi^) CD4 and CD8 T cells were reduced by approximately 40% and 30%, respectively in spleen, whereas the numbers of effector memory T cells (CD44^hi^ and CD62L^lo^) were not affected (Fig. 4B–C). Therefore, peripheral naive T cells were specifically reduced in the absence of Rasal3. Because development in the thymus was normal, survival of mature T cell might be impaired in Rasal3 deficient mice. As shown in Fig. 4D and E, the frequency of Annexin V positive cells (apoptotic cells) was significantly increased in Rasal3 deficient naive CD44^lo^ T cells. Death of CD4 T cells was more significant than that of CD8 T cells, and CD4 cells double positive for Annexin V and 7AAD (necrotic cells) were also increased. Conversely, the frequency of apoptotic cells in the CD4^-^ CD8^-^ and TCRβ^-^ population (mainly B cells) was not increased. Collectively, these results suggest that Rasal3 is important for survival of mature naive T cells.

### Survival of naive T cells in vivo was dependent on Rasal3

**Fig 5:**
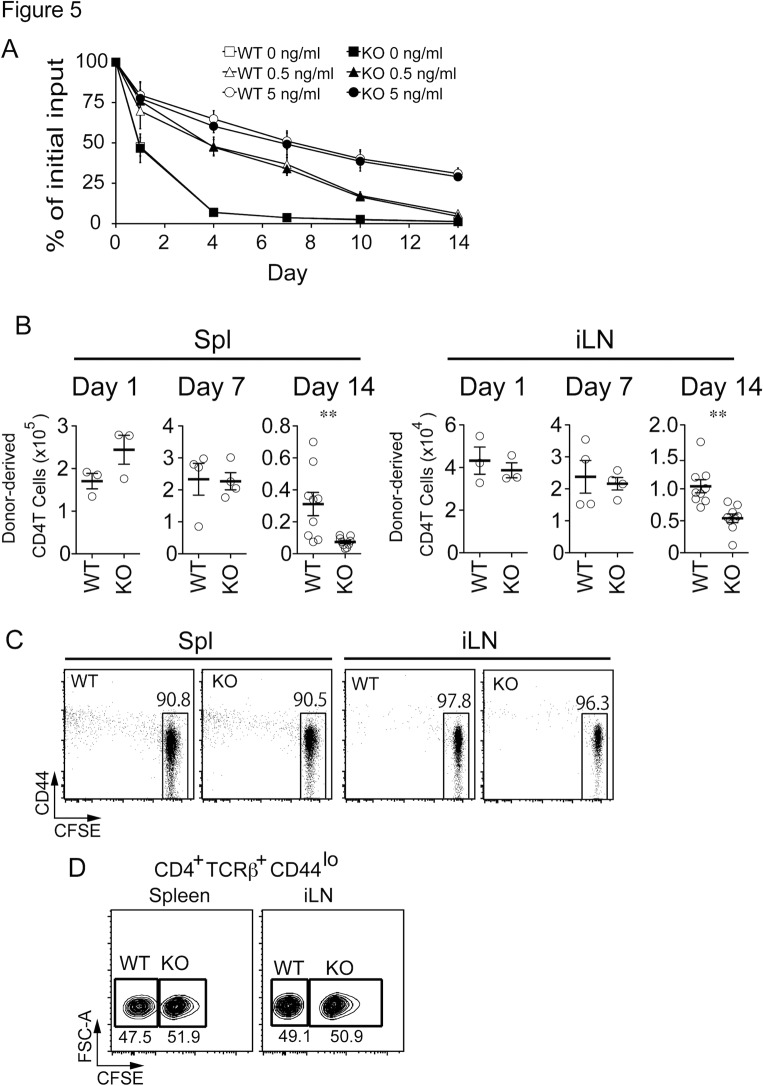
Impaired survival of Rasal3 *in vivo* but not *in vitro*. (A) Isolated splenic CD4 T cells (CD4^+^ CD25^-^ CD44^lo^ CD62L^hi^) were cultured with or without IL-7. IL-7 was added into culture medium at the final concentration of 0 (square), 0.5 (triangle) and 5 (circle) ng/ml. Cell viability was normalized to initial live cell number. (B) Impaired cell survival of Rasal3-deficient naive T cells *in vivo*. CD4^+^ TCRβ^+^ CD44^lo^ cells (3 x 10^6^) from CD45.2 WT or Rasal3-KO mice were intravenously injected into CD45.1 congenic mice. At 1, 7 and 14 days after transfer, the numbers of donor-derived (CD45.2^+^) CD4^+^ TCRβ^+^ cells in spleen and iLN were determined. (C) Most of transferred cells remained undivided in CD45.1 congenic mice. CFSE-labeled CD4^+^ TCRβ^+^ CD44^lo^ cells (3 x 10^6^) were intravenously injected into CD45.1 mice. At 14 days after transfer, CFSE dilution and CD44 expression in CD4^+^ TCRβ^+^ CD45.2^+^ cells in spleen and iLN were analyzed. (D) Migration ability is normal in Rasal3-deficient naive T cells. CD4^+^ TCRβ^+^ CD44^lo^ cells from WT (non-labeled) and Rasal3-KO mice (CFSE-labeled) were mixed at the ration of 1:1 and intravenously injected into CD45.1 mice. At 1 hour after transfer, proportion of WT and Rasal3-deficient T cells in spleen and iLN of recipient mice was analyzed.

Because apoptosis of peripheral naive T cells was increased in Rasal3 deficient mice, survival of Rasal3-deficient T cells was first examined *in vitro*. It is known that IL-7 is important for maintenance of naive T cell survival [3]. Therefore, CD25^-^, CD62L^hi^, CD44^lo^ naive CD4 T cells were cultured in complete medium with or without IL-7 at a final concentration of 0.5 and 5.0 ng/ml. As shown in Fig. 5A, IL-7 enhanced the survival of naive CD4 T cells *in vitro*, however, no difference was observed between the Rasal3 deficient and wild-type (WT) cells, indicating that Rasal3 is not related to the IL-7-mediated survival signal. Next, to examine the effects of Rasal3 deficiency on cell survival *in vivo*, sorted naive CD25^-^ CD44^lo^ CD4^+^ cells were intravenously injected into CD45.1^+^ congenic B6 mice, and the number of donor-derived CD45.2^+^ TCRβ^+^ CD4^+^ cells in the spleen and iLN after transfer was counted. Although the absolute number of transferred T cells was not changed in the absence of Rasal3 up to 7 days, it was significantly decreased at 14 days after transfer (Fig. 5B). These transferred T cells still showed a naive phenotype and had not experienced proliferation, because even 2 weeks after transfer, over 90% of donor derived T cells remained undivided, by the detection of carboxyfluorescein diacetate succinimidyl ester (CFSE) dilution (Fig. 5C). The reduction of transferred T cells in the spleen and iLN could be because of differential distribution to the various secondary lymphoid organs, therefore, we investigated the homing ability of Rasal3-dificient T cells to the spleen and iLN. As shown in Fig. 5D, homing of injected naive T cells to the spleen and iLN was equivalent in Rasal3 deficient T cells, excluding the possibility of aberrant homing. Because both proliferation and homing ability were normal in Rasal3-deficient naive T cells, we concluded that survival of the Rasal3-deficient naive T cells *in vivo*, but not *in vitro* was impaired.

## Discussion

In the current study, we characterized Rasal3, a novel member of the RasGAP Rasal family, as being predominantly expressed in T lineage cells and important for survival of naive T cells in the periphery.

As predicted from its primary structure, Rasal3 possesses RasGAP activity, and Rasal3 overexpressing DPK cells showed both reduced accumulation of GTP-bound Ras and phosphorylation of ERK upon TCR stimulation (Fig. 1D). Certain GAP proteins reportedly show dual/multiple substrate specificity; for example, Rasa3 has dual GAP activity both to Ras and Rap1 [25]. Since Rasal3 harbors a proline at position 641 (S1A Fig.), which corresponds to proline 489 in Rasa3, and important for its Rap1GAP activity [25], we assumed that Rasal3 might also have Rap1GAP activity. However, TCR-dependent accumulation of GTP-bound Rap1 was unaffected by overexpression of Rasal3 (S1B Fig.). Therefore, we concluded that Rasal3 does not have GAP activity against Rap.

Since Ras is a membrane-associated GTP-binding protein [16], RasGAP should be localized proximal to the plasma membrane to activate Ras GTPase activity. As Rasal3 protein is located mainly in the cytoplasm, Rasal3 may translocate to the plasma membrane to fulfill its function. Indeed, Rasal1, another Rasal family member, was shown to translocate to the plasma membrane in a calcium-dependent manner [27]. Rasal3 has a C2 domain, which is involved in Calcium-dependent membrane localization, and a PH domain, which can bind phosphatidylinositol lipids in cell membrane, suggesting the possibility that Rasal3 can translocate to the plasma membrane by TCR-induced calcium influx and phosphoinositide generation. However, we did not observe translocation of Rasal3 protein into the membrane fraction upon TCR stimulation (data not shown). Since Rasal3 was reported to be serine-phosphorylated upon TCR stimulation by phosphoproteomic analysis [28], post-translational modification of Rasal3 could be important for association with Ras.

Involvement of two members of thymus-expressed RasGAPs, Rasa1 and Neurofibromin 1 (NF1), in T cell development has been previously reported. Rasa1 negatively regulates TCR-induced phosphorylation of ERK, consistent with its function as a RasGAP. Although positive selection of polyclonal TCRs was not affected in Rasa1 deficient thymus, positive selection of monoclonal-TCR in AND-TCR Tg mice was enhanced [17]. On the contrary, NF1 did not show inhibitory effects on TCR-induced activation of Ras/MAPK. Positive selection of polyclonal TCR was not affected in NF1 deficient mice, but surprisingly, positive selection of monoclonal HY-TCR was reduced in the absence of NF1 [18], which was the opposite result predicted for a RasGAP. The authors suggested that a function other than RasGAP activity may be responsible for the inhibitory effect on the positive selection of HY thymocytes. Collectively, deletion of either one of these two thymic RasGAPs did not show prominent effects on global development of T cells in the thymus. In the current study, Rasal3-deficient mice showed normal T cell development on a wild-type (polyclonal) background as well as in OT-I and OT-II TCR-transgenic mice. Thus, unlike Rasa1 and NF1, Rasal3 was dispensable for T cell development in the thymus. As all these RasGAPs likely have overlapping redundant roles as well as independent specific functions, mice doubly or triply deficient for Rasal3, Rasa1, and NF1 may be needed for fully understanding the cooperative roles of RasGAPs in T cell development.

While T cell development in the thymus was unaffected, the number of peripheral naive CD4 and CD8 T cells in spleen was significantly reduced in Rasal3-deficient mice (Fig. 4B–C). This reduction was observed only in naive T cell populations but not in activated memory T cells, indicating that activation and transition to memory phenotype T cells occurs normally in the absence of Rasal3. In fact, Rasal3-deficient naive T cells showed a greater susceptibility to apoptosis as evidenced by the increased frequency of Annexin V positive T cells in Rasal3-deficient mice (Fig. 4D–E). However, survival of isolated naive T cells during long-term culture (up to 14 days) with or without IL-7 *in vitro* was unaltered between WT and Rasal3-deficient mice (Fig. 5A), indicating that Rasal3 is not related to IL-7-mediated survival signaling.

Despite the normal survival of naive T cells *in vitro*, the number of Rasal3-deficient naive CD4 T cells adoptively transferred into WT mice was significantly impaired (Fig. 5B). These results, along with the data that proliferation and migration of transferred T cells were unaffected, suggest that Rasal3 is important for *in vivo* survival of naive T cells. One of the apparent differences between survival *in vivo* and *in vitro* is the existence of tonic TCR signals. Survival of mature naive T cells in the periphery requires weak continuous TCR signaling, called tonic TCR signaling [2–3], because various reports showed a critical requirement for interaction of TCR with self peptide-class I and II MHC for survival of naive T cells [5–8]. It is therefore possible that Rasal3 plays a role in tonic TCR signaling for maintenance of T cell survival.

In the current study, we did not observe augmentation of TCR-dependent ERK activation in Rasal3 deficient T cells (Fig. 3), indicating that TCR-dependent activation of Ras was not increased. Indeed, we did not observe any sign of augmented TCR-dependent signal transduction such as Ca^2+^ influx, CD69-upregulation, IL-2 production, and proliferation (Fig. 3C-G) in Rasal3 deficient T cells, probably because of compensatory effects of other redundant RasGAPs in T cells. Although anti-TCR antibody-induced strong activation of Ras was not affected by Rasal3 deficiency, it is still possible that Rasal3 regulates Ras activity only when TCR signal strength is weak enough to induce tonic TCR stimulation. If this is the case, tonic TCR signaling would be augmented in Rasal3 deficient T cells. The sevenmaker ERK2-Tg mice, which express a dominant gain-of-function mutant of ERK2, exhibited reduced numbers of peripheral CD8 naive T cells, suggesting that increased ERK activation could inhibit tonic survival signals [29]. Therefore, one possible explanation is that enhanced Ras-MAPK activation by the lack of Rasal3 dysregulates tonic TCR signals, resulting in impaired cell survival of naive T cells. Interestingly, mice deficient for Rasa1 and NF1, two other RasGAPs in T cells, also exhibited impaired survival of peripheral mature T cells [17–18], although they had different effects on positive selection in the thymus. Therefore, RasGAPs, including Rasal3, play critical roles in the survival of naive T cells. It is also possible that Rasal3 has an additional function other than GAP activity for promoting naive T cell survival.

Taken together, we are the first to characterize Rasal3, a newly described member of the Rasal family, as an active RasGAP expressed in T lineage cells and essential for *in vivo* survival of naive T cells and maintenance of the peripheral T cell pool in the body. Further experiments are needed to fully understand the precise molecular mechanisms of Rasal3 regulation of *in vivo* T cell survival, including how Rasal3 cooperates with other RasGAPs expressed in T cells.

## Supporting Information

S1 Fig Rasal3 is not a Rap1GAP. (PDF)

S2 Fig Generation of Rasal3 deficient mice. (PDF)

S3 Fig The number and frequency of thymic cell subpopulations. (PDF)

S4 Fig The number and frequency of peripheral T cells. (PDF)

S5 Fig Full length images of immunoblots. (PDF)

## References

[1] Rothenberg EV, Taghon T. Molecular genetics of T cell development. Anne Rev Immunol. 2005;23: 601–649. 15771582 10.1146/annurev.immunol.23.021704.115737

[2] Boyman O, Létourneau S, Krieg C, Sprent J. Homeostatic proliferation and survival of naive and memory T cells. Eur J Immunol. 2009;39:2088–2094. 10.1002/eji.200939444 19637200

[3] Sprent J, Surh CD. Normal T cell homeostasis: the conversion of naive cells into memory-phenotype cells. Nat Immunol. 2011;12:478–484. 21739670 10.1038/ni.2018 PMC3434123

[4] Tanchot C, Lemonnier FA, Pérarnau B, Freitas AA, Rocha B. Differential requirements for survival and proliferation of CD8 naive or memory T cells. Science. 1997;276:2057–2062. 9197272 10.1126/science.276.5321.2057

[5] Murali-Krishna K, Lau LL, Sambhara S, Lemonnier F, Altman J, Ahmed R. Persistence of memory CD8 T cells in MHC class I-deficient mice. Science. 1999;286:1377–1381. 10558996 10.1126/science.286.5443.1377

[6] Takeda S, Rodewald HR, Arakawa H, Bluethmann H, Shimizu T. MHC class II molecules are not required for survival of newly generated CD4^+^ T cells, but affect their long-term life span. Immunity. 1996;5:217–228. 8808677 10.1016/s1074-7613(00)80317-9

[7] Rooke R, Waltzinger C, Benoist C, Mathis D. Targeted complementation of MHC class II deficiency by intrathymic delivery of recombinant adenoviruses. Immunity. 1997;7:123–134. 9252125 10.1016/s1074-7613(00)80515-4

[8] Dorfman JR, Stefanová I, Yasutomo K, Germain RN. CD4^+^ T cell survival is not directly linked to self-MHC-induced TCR signaling. Nat Immunol. 2004;1:329–335. 10.1038/79783 11017105

[9] Grandjean I, Duban L, Bonney EA, Corcuff E, Di Santo JP, Matzinger P, et al Are major histocompatibility complex molecules involved in the survival of naive CD4^+^ T cells? J Exp Med. 2003;198:1089–1102. 14517277 10.1084/jem.20030963 PMC2194222

[10] Kim K, Lee CK, Sayers TJ, Muegge K, Durum SK The trophic action of IL-7 on pro-T cells: inhibition of apoptosis of pro-T1,-T2, and -T3 cells correlates with Bcl-2 and Bax levels and is independent of Fas and p53 pathways. J Immunol 1998;160:5735–5741 9637482

[11] Li WQ, Jiang Q, Khaled AR, Keller JR, Durum SK. Interleukin-7 inactivates the pro-apoptotic protein Bad promoting T cell survival. J Biol Chem 2004;279:29160–29166. 15123689 10.1074/jbc.M401656200

[12] Kortum RL, Rouquette-Jazdanian AK, Samelson LE. Ras and extracellular signal-regulated kinase signaling in thymocytes and T cells. Trends Immunol 2013;34:259–268. 10.1016/j.it.2013.02.004 23506953 PMC3856398

[13] Crompton T, Gilmour KC, Owen MJ. The MAP kinase pathway controls differentiation from double-negative to double-positive thymocyte. Cell. 1996;26:243–251. 10.1016/s0092-8674(00)80096-3 8706129

[14] Alberola-Ila J, Hernández-Hoyos G. The Ras/MAPK cascade and the control of positive selection. Immunol Rev. 2003; 191:79–96. 12614353 10.1034/j.1600-065x.2003.00012.x

[15] Kortum RL, Sommers CL, Pinski JM, Alexander CP, Merrill RK, Li W, et al Deconstructing Ras signaling in the thymus. Mol Cell Biol. 2012;32:2748–2759. 10.1128/MCB.00317-12 22586275 PMC3416180

[16] King PD, Lubeck BA, Lapinski PE. Nonredundant functions for Ras GTPase-activating proteins in tissue homeostasis. Sci Signal. 2013;6:re1 10.1126/scisignal.2003669 23443682 PMC5483993

[17] Lapinski PE, Qiao Y, Chang CH, King PD. A role for p120 RasGAP in thymocyte positive selection and survival of naive T cells. J Immunol. 2011;187:151–163. 10.4049/jimmunol.1100178 21646295 PMC3119767

[18] Oliver JA, Lapinski PE, Lubeck BA, Turner JS, Parada LF, Zhu Y, et al The Ras GTPase-activating protein neurofibromin 1 promotes the positive selection of thymocytes. Mol Immunol. 2013;55:292–302. 10.1016/j.molimm.2013.03.005 23522726 PMC3646930

[19] Patrick MS, Oda H, Hayakawa K, Sato Y, Eshima K, Kirikae T, et al Gasp, a Grb2-associating protein, is critical for positive selection of thymocytes. Proc Natl Acad Sci U S A. 2009;106:16345–1650. 10.1073/pnas.0908593106 19805304 PMC2752560

[20] Oda H, Fujimoto M, Patrick MS, Chida D, Sato Y, Azuma Y, et al RhoH plays critical roles in Fc epsilon RI-dependent signal transduction in mast cells. J Immunol. 2009;182:957–962. 19124738 10.4049/jimmunol.182.2.957

[21] Kisielow P, Teh HS, Blüthmann H, von Boehmer H. Positive selection of antigen-specific T cells in thymus by restricting MHC molecules. Nature. 1998;335:730–733. 10.1038/335730a0 3262831

[22] Shinkai Y, Koyasu S, Nakayama K, Murphy KM, Loh DY, Reinherz EL, et al Restoration of T cell development in RAG-2-deficient mice by functional TCR transgenes. Science. 1991;259:822–825. 10.1126/science.8430336 8430336

[23] Nitta T, Ohigashi I, Takahama Y. The development of T lymphocytes in fetal thymus organ culture. Methods Mol Biol. 2013;946:85–102. 10.1007/978-1-62703-128-8_6 23179827

[24] Saitoh T, Nakano H, Yamamoto N, Yamaoka S. Lymphotoxin-beta receptor mediates NEMO-independent NF-kappaB activation. FEBS Lett. 2002;532:45–51. 12459460 10.1016/s0014-5793(02)03622-0

[25] Kupzig S, Bouyoucef-Cherchalli D, Yarwood S, Sessions R, Cullen PJ. The ability of GAP1IP4BP to function as a Rap1 GTPase-activating protein (GAP) requires its Ras GAP-related domain and an arginine finger rather than an asparagine thumb. Mol Cell Biol. 2009;29:3929–3940. 10.1128/MCB.00427-09 19433443 PMC2704752

[26] Kisielow P, Blüthmann H, Staerz UD, Steinmetz M, von Boehmer H. Tolerance in T-cell-receptor transgenic mice involves deletion of nonmature CD4^+^8^+^ thymocytes. Nature. 1988;333:742–746. 3260350 10.1038/333742a0

[27] Walker SA, Kupzig S, Bouyoucef D, Davies LC, Tsuboi T, Bivona TG, et al Identification of a Ras GTPase-activating protein regulated by receptor-mediated Ca^2+^ oscillations. EMBO J. 2004;23:1749–1760. 15057271 10.1038/sj.emboj.7600197 PMC394250

[28] Navarro MN, Goebel J, Feijoo-Carnero C, Morrice N, Cantrell DA. Phosphoproteomic analysis reveals an intrinsic pathway for the regulation of histone deacetylase 7 that controls the function of cytotoxic T lymphocytes. Nat Immunol. 2011;12:352–361. 10.1038/ni.2008 21399638 PMC3110993

[29] Sharp LL, Schwarz DA, Bott CM, Marshall CJ, Hedrick SM. The influence of the MAPK pathway on T cell lineage commitment. Immunity. 1997;7:609–618. 9390685 10.1016/s1074-7613(00)80382-9

