# Supplementary material for: The Ras GTPase-Activating Protein Rasal3 Supports Survival of Naive T Cells
Source: PLoS One. 2015 Mar 20;10(3):e0119898. doi: 10.1371/journal.pone.0119898 (PMC4368693; doi:10.1371/journal.pone.0119898)
Supplement: S1 Fig — (PDF) [file pone.0119898.s001.pdf]

Supporting Infomation 1 Muro et al.

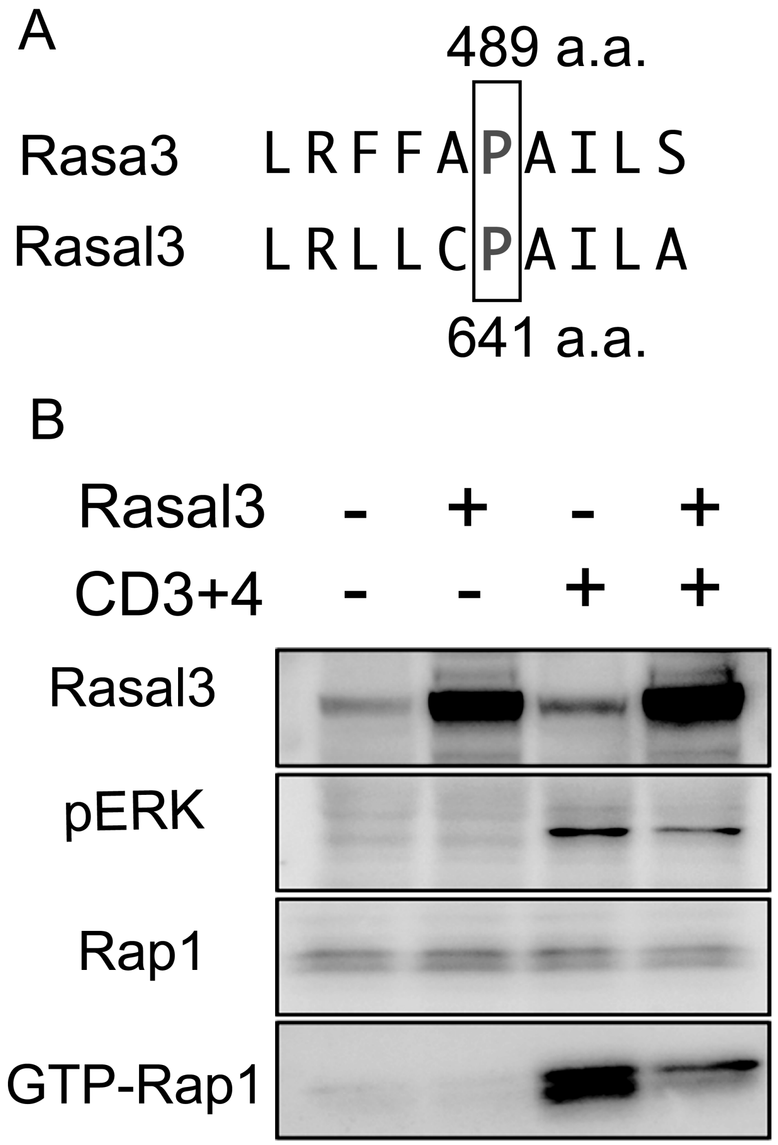

(A) Comparison of amino acid sequence between Rasa3 and Rasal3. (B) Analysis of Rasal3 Rap1GAP function. A Rasa3 overexpressing DPK cell line was used for affinity precipitation assay of GTP-Rap1. The results are representative of more than three independent experiments.
