# Supplementary material for: The Ras GTPase-Activating Protein Rasal3 Supports Survival of Naive T Cells
Source: PLoS One. 2015 Mar 20;10(3):e0119898. doi: 10.1371/journal.pone.0119898 (PMC4368693; doi:10.1371/journal.pone.0119898)
Supplement: S2 Fig — (PDF) [file pone.0119898.s002.pdf]

Supporting Infomation 2 Muro et al.

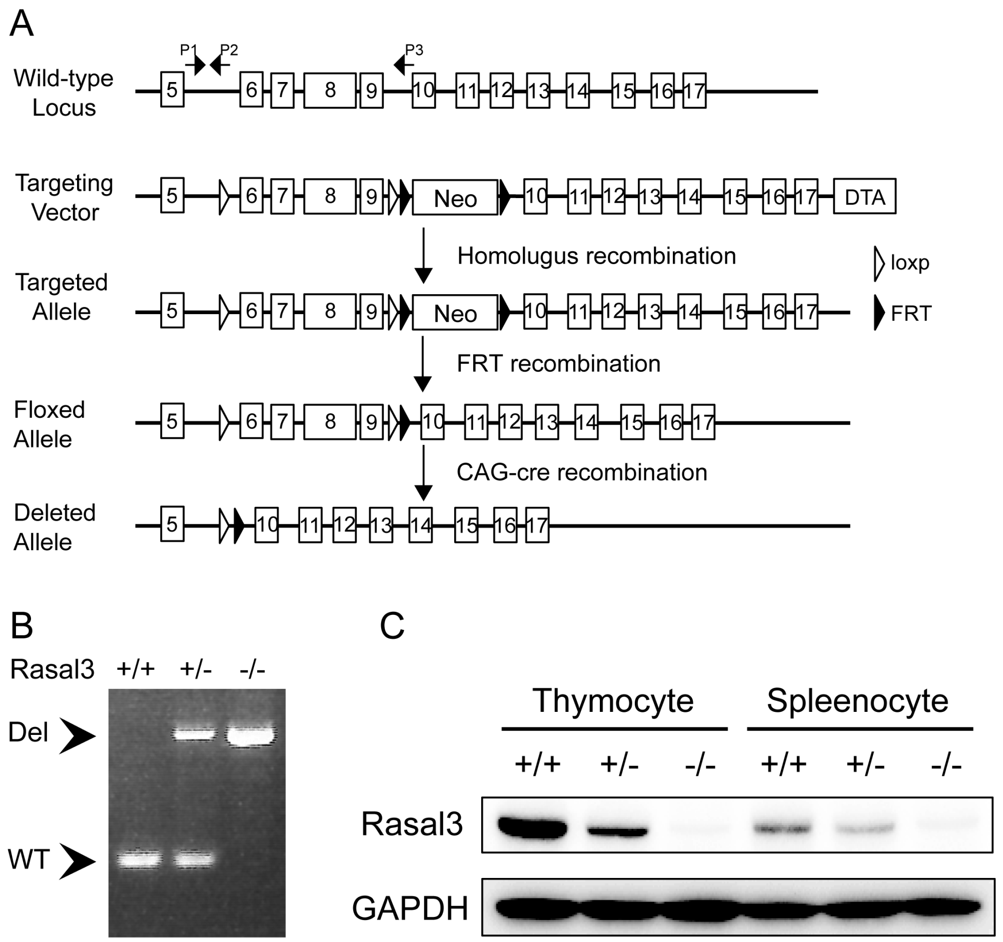

(A) Schematic diagram of Rasal3-deficient loci was shown. Generation of Rasal3-mice was described in Materials and Methods in detail. (B) Confirmation of Rasal3 deficiency by PCR. To detect mice genotypes, the following primers were used ; P1 TCTATTCTGCTTTATGCTAGGTGAAC, P2 ATCCTAGTGATTGATCCGCTATTTC, P3 CAATCAAGTTTGAGTGCCCTGC. (C) Confirmation of Rasal3 deficiency by Western blot.
