# Supplementary material for: The Ras GTPase-Activating Protein Rasal3 Supports Survival of Naive T Cells
Source: PLoS One. 2015 Mar 20;10(3):e0119898. doi: 10.1371/journal.pone.0119898 (PMC4368693; doi:10.1371/journal.pone.0119898)
Supplement: S3 Fig — (PDF) [file pone.0119898.s003.pdf]

Supporting Infomation 3 Muro et al.

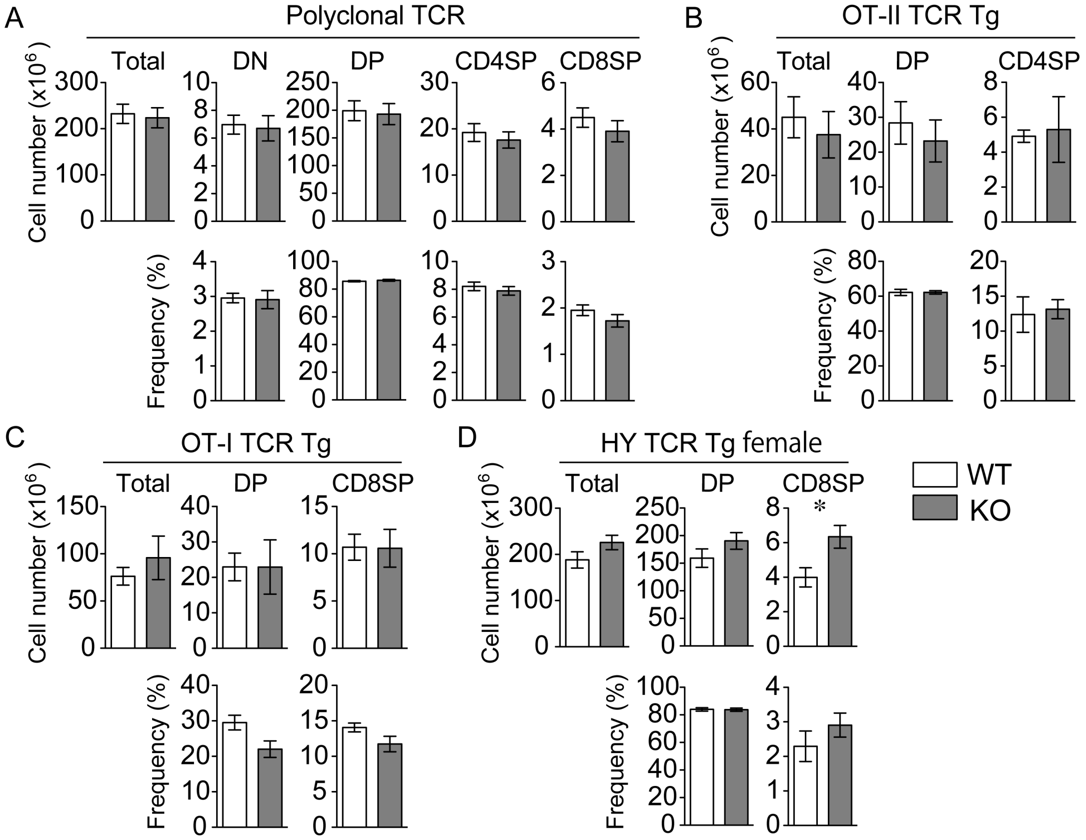

(A), (B), (C) and (D) show cell number and frequency of thymic cell subpopulation in poly clonal TCR, OT-II TCR Tg, OT-I TCR Tg and HY TCR Tg mice, respectively. All TCR Tgs were on a $Rag^{-/-}$ background.
