# Supplementary material for: The Ras GTPase-Activating Protein Rasal3 Supports Survival of Naive T Cells
Source: PLoS One. 2015 Mar 20;10(3):e0119898. doi: 10.1371/journal.pone.0119898 (PMC4368693; doi:10.1371/journal.pone.0119898)
Supplement: S4 Fig — (PDF) [file pone.0119898.s004.pdf]

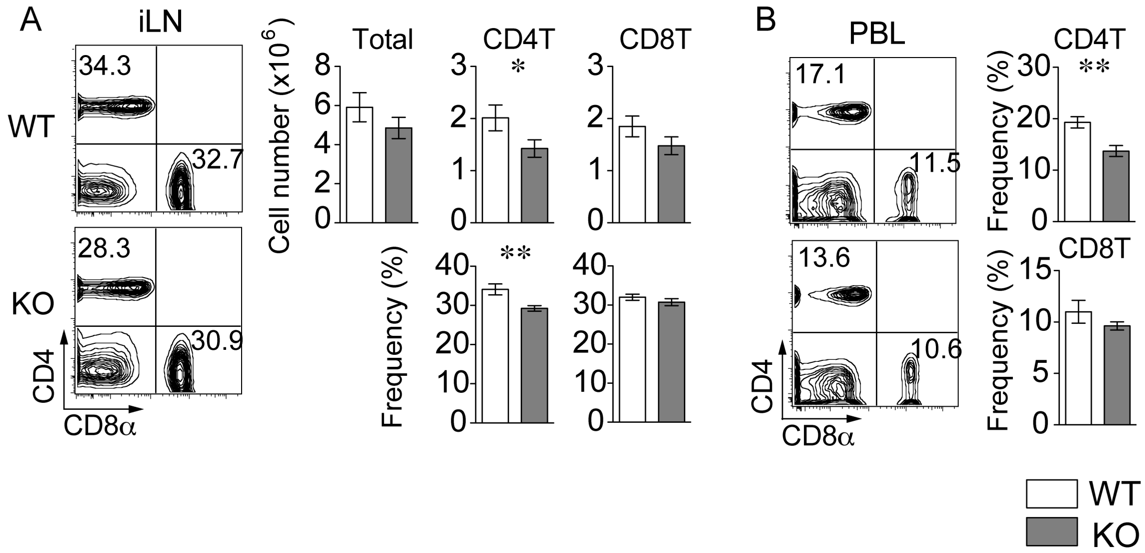

(A) CD4 and CD8 profile of inguinal lymph node cells from WT and Rasal3-KO mice.

(B) CD4 and CD8 profile of peripheral blood lymphocytes from WT and Rasal3-KO mice.
