# Supplementary material for: The Ras GTPase-Activating Protein Rasal3 Supports Survival of Naive T Cells
Source: PLoS One. 2015 Mar 20;10(3):e0119898. doi: 10.1371/journal.pone.0119898 (PMC4368693; doi:10.1371/journal.pone.0119898)
Supplement: S5 Fig — (PDF) [file pone.0119898.s005.pdf]

Supporting Infomation 5 Muro et al.

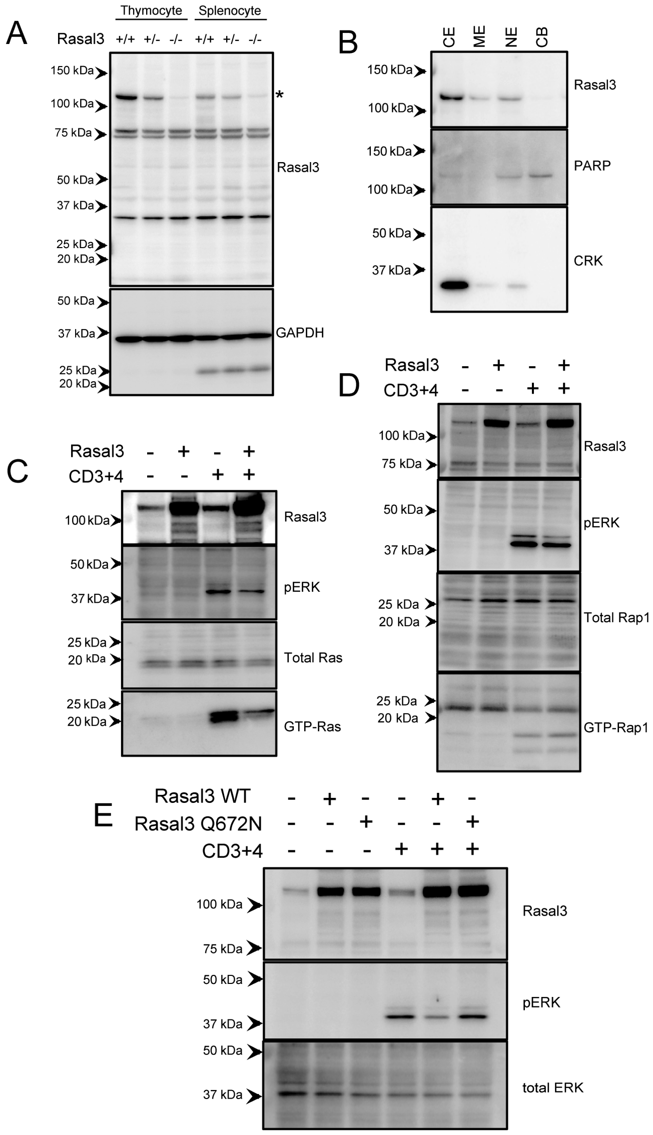

(A) Western blot with Anti-Rasal3 and GAPDH were shown. An asterisk indicates Rasal3 signal band. (B) Subcellular localization of Rasal3 protein in thymocytes, related to Fig. 1C. (C) RasGAP analysis, related to Fig. 1D. (D) Rap1GAP analysis, related to Fig. S1. (E) Rasal3 Q672N mutant loses RasGAP activity, related to Fig. 1E.
